# Chemical Characterization and Biological Activities of a Beverage of *Zuccagnia punctata*, an Endemic Plant of Argentina with Blueberry Juice and Lemon Honey

**DOI:** 10.3390/plants13223143

**Published:** 2024-11-08

**Authors:** Florencia María Correa Uriburu, Iris Catiana Zampini, Luis María Maldonado, Milagros Gómez Mattson, Daniela Salvatori, María Inés Isla

**Affiliations:** 1 Instituto de Bioprospección y Fisiología Vegetal (INBIOFIV), CONICET—Universidad Nacional de Tucumán (UNT), San Martín 1545, San Miguel de Tucumán T4000CBG, Argentina; florcorreau@gmail.com (F.M.C.U.); zampini@csnat.unt.edu.ar (I.C.Z.); 2 Instituto Nacional de Tecnología Agropecuaria, Estación Experimental Agropecuaria, Famaillá (INTA), Ruta Provincial 301-km 32, Famaillá 4132, Argentina; lmaldo@correo.inta.gov.ar; 3 Facultad de Ciencias Naturales e Instituto Miguel Lillo (UNT), Universidad Nacional de Tucumán, Miguel Lillo 205, San Miguel de Tucumán T4000JFE, Argentina; 4 Instituto de Investigación y Desarrollo en Ingeniería de Procesos, Biotecnología y Energías Alternativas (PROBIEN), Neuquén 8300, Argentina; milagros.gomez@probien.gob.ar (M.G.M.); dmsalvatori@hotmail.com (D.S.)

**Keywords:** *Zuccagnia punctata*, *Vaccinium corymbosum*, functional beverages, powder

## Abstract

In this study, the production of functional beverages of *Zuccagnia punctata* Cav. (jarilla), a native medicinal plant from Argentina, and *Vaccinium corymbosum* (blueberry), with lemon honey as a sweetener, is described. The beverage was formulated by using jarilla extract and blueberry juice with maltodextrin as an encapsulant material. The beverage was dried by both spray-drying and freeze-drying. Both beverages showed high water solubility with adequate features for handling, transport, and storage. The chromatic parameters indicate tones of mauve. Both the total polyphenol and flavonoid contents were retained after being spray-dried (92 and 100%, respectively). The anthocyanins were less stable under spray-dried conditions (58% retained). Both beverages showed high scavenger capacity on ABTS^•+^, HO^•^, and H_2_O_2_ (SC_50_ between 3.56 and 36.90 µg GAE/mL) and exhibited in vitro inhibitor potential of α-glucosidase, α-amylase, and lipase activities (IC_50_ of between 2.97 and 27.19 µg GAE/mL). The powdered beverage obtained by spray-drying presented the greatest preference in sensory tests. The beverages were neither toxic nor mutagenic in the concentration range with biological activity. During short-term storage, both beverages showed stability. The results obtained would support the use of a powdered beverage made from an Argentinean native plant and blueberries as a functional food.

## 1. Introduction

Beverages are a convenient way to consume substances with functional properties. Functional beverages contain bioactive components, i.e., phenolic compounds, minerals, vitamins, amino acids, peptides, unsaturated fatty acids, and other compounds with biological properties [1]. To improve consumer perception and enhance the stability of the active ingredients, encapsulation by spray-drying or freeze-drying, emulsification, and certain high-pressure homogenization procedures are used [2]. Numerous materials, which include proteins, gums, and modified starches, have been used as encapsulating agents [2]. Maltodextrin (MD) is a partially hydrolyzed starch that has been frequently used as a reference wall material for encapsulation [2]. The present research focuses on the development and characterization of a powdered functional beverage of *Zuccagnia punctata* extract with *Vaccinium corymbosum* juice, by applying a spray-drying and freeze-drying process.

**Figure 1:**
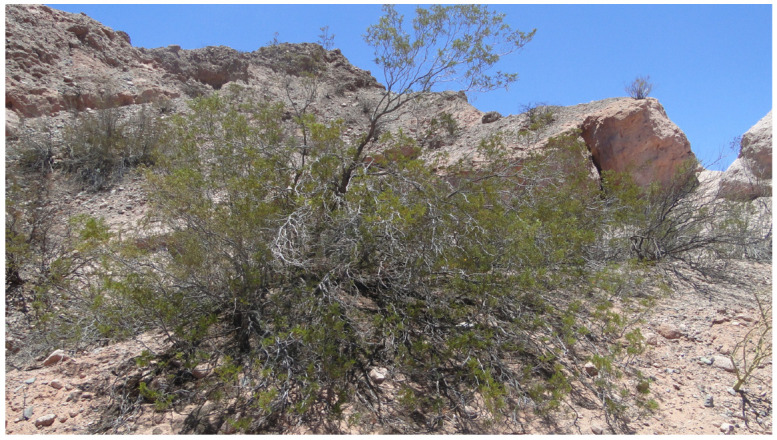
Photography of *Zuccagnia punctata* Cav., at the collection site (Amaicha del Valle, Tucumán, Argentina).

*Zuccagnia punctata* Cav. (common name: jarilla) is an Argentine endemic aromatic shrub (Figure 1) popularly used as a medicinal plant [3]. Several extract types of *Z. punctata* have been involved in a wide range of biological activities, i.e., antibacterial, antifungal, nematicide, cytoprotective, anti-inflammatory, antioxidant, antimutagenic, and antitumoral [4,5,6,7,8,9,10,11,12,13,14,15,16,17,18,19]. Oral administration of either *Z. punctata* ethanolic extract or some of its isolated compounds has a significant effect on the prevention of cardiovascular diseases related to hypercholesterolemia, hyperglycemia, and endothelial dysfunction [20,21,22,23]. The main bioactive specialized metabolites in *Z. punctata* leaves were identified as 2′,4′-dihydroxy-3′-methoxy chalcone (DHMC) and 2′,4′-dihydroxychalcone (DHC), proposed as bioactive chemical markers [2,3,4,5,6,7,8,9,10,11,12,13,14,15,16,17,18,19,20,21,22,23].

The berry fruits are fleshy, juicy, and bittersweet. They provide foods with both attractive colors and chemical components beneficial to health. The fruits of *Vaccinium corymbosum* L. have been extensively studied in terms of their chemical composition, as well as their biological and functional properties. Chemical studies revealed that the berries are rich in fiber, potassium, ascorbic acid, anthocyanins, and other phenolic compounds, such as flavonols, flavanols, and phenolic acids [24,25,26,27,28]. Several beneficial health activities such as antioxidant, antiobesity, and antimicrobial [24,25,26,27] have been reported. On the other hand, the research of Carvalho et al. (2021) [28] on processes associated with metabolic syndrome revealed that the consumption of blueberries reduced total cholesterol and low-density lipoprotein levels, as well as diastolic blood pressure. The properties previously demonstrated for both plant species make them very promising for the formulation of functional beverages. Therefore, the aim of the present study was to obtain a powder beverage of *Z. punctata* extract and *V. corymbosum* fruit juice so as to characterize it chemically and determine its functional properties.

## 2. Results and Discussion

The aqueous and ethanolic extracts of *Z. punctata* and blueberries have shown important functional properties in pathologies related to oxidative stress. For this reason, a non-alcoholic beverage containing phytochemical or specialized metabolites obtained from dry aerial parts of *Z. punctata* in ethanol 5% and blueberry juice was prepared, and lemon honey was added as a sweetener. The solvent used was selected according to recommendations of the Argentine Food Code, Chapter XII, Article 996, dealing with non-alcoholic beverages [29].

### 2.1. Chemical Characterization of Z. punctata Extract and Blueberry Juice

**Figure 2:**
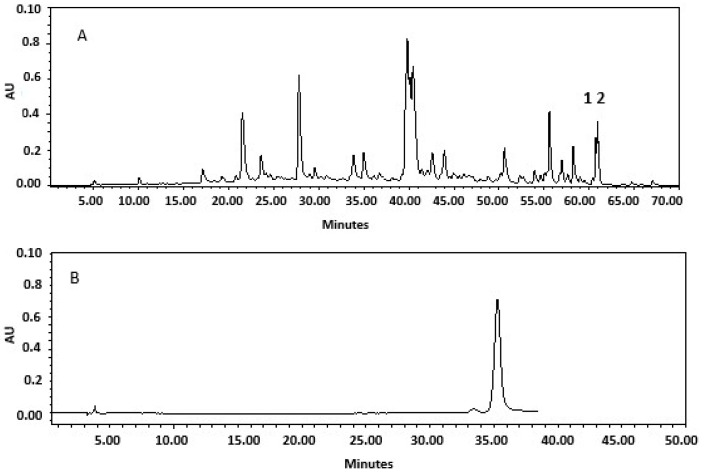
HPLC DAD profile of (**A**) *Z. punctata* extract showing (1) 2′,4′-dihydroxychalcone (DHC), and (2) 2′,4′-dihydroxy-3′-methoxy chalcone (DHMC) and (**B**) hesperidin in lemon honey.

The extract in ethanol 5% of *Z. punctata* aerial parts was chemically characterized: 0.6% total sugar; 0.6% total phenolic compounds; 1.2% total flavonoids; and 3.8% condensed tannins. The tannins are responsible for the astringent flavor of *Z. punctata* extract. Chalcones such as DHC (2.3 mg/100 mL) and DHMC (1.2 mg/100 mL), two marker metabolites of *Z. punctata,* were also quantified (Figure 2). Anthocyanins were not detected in the *Z. punctata* extract. The blueberry juice contained 13% reducing sugar. Sucrose was not detected. Specialized metabolites of blueberry juice were mainly 0.11% phenolic compounds, 0.16% flavonoids, 0.51% hydrolyzed tannins, 0.12% condensed tannins, and 0.04% total anthocyanins. The HPLC profile of the lemon honey showed a characteristic peak of a chemical marker of this honey, corresponding to hesperidin, and it was quantified (6 mg/kg) (Figure 2).

### 2.2. Formulation and Chemical Characterization of a Beverage Made from Z. punctata and Blueberries

This is the first time that a beverage containing 9% *Z. punctata* extract and 27% blueberry juice, sweetened [29] with lemon honey (12 °Brix of soluble solids), has been developed. The resulting beverage contained total and reducing sugar, flavonoids, and phenolic compounds. The chemical composition of the beverage, formulated before being subjected to a drying process, is shown in Table 1.

**Figure 3:**
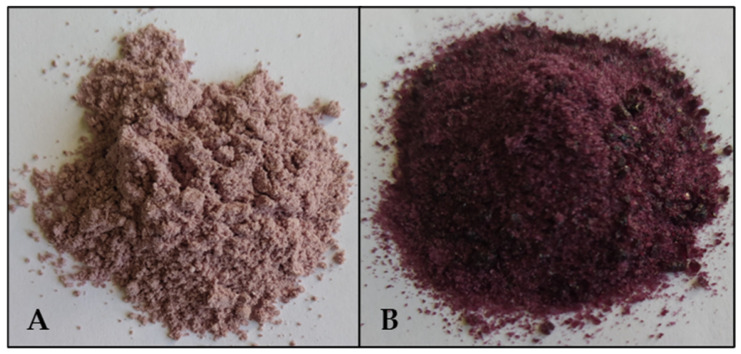
Beverages dried by (**A**) spray-drying; (**B**) freeze-drying.

A powdered beverage with low water activity (a_w_) is convenient because it is easy to transport and store, not to mention the fact that it is chemically and microbiologically stable. The formulated *Z. punctata* beverage in the present study was subjected to a drying process by freeze-drying and spray-drying to obtain powdered beverages (Figure 3) and was microencapsulated with 15% MD DE-15 [30,31]. The yield of the beverage achieved was 92% for freeze-drying and 45% for spray-drying. The low performance in spray-drying may be due to the fact that only part of the powder is recovered, whereas the rest remains on the walls of the dryer and in the cyclone. In studies conducted by Tonón et al. (2009) [32], açai fruits were microencapsulated in MD by spray-drying, and the yield did not exceed 48.4%. In the case of drying *Morinda citrifolia* L. with different concentrations of MD, the yield ranged between 48.1 and 51% [33]. Similarly, in encapsulation studies of *Vaccinium myrtillus* juice using another encapsulation material (cyclodextrins) and spray-drying, a yield of 46.2% was achieved; with maltodextrin (MD), a yield of 45.9% was reported [34]. So, the yield obtained for the powdered beverage of *Z. punctata* by spray-drying could be considered adequate, while that produced by freeze-drying was excellent.

The powdered beverages were chemically characterized. The soluble monosaccharides present in the powdered beverages were fructose and glucose in both drying methods (Table 2). In both powders, the concentration of fructose was higher than that of glucose and no quantifiable levels of sucrose were detected. The protein content in both powders was quite low. Organic acids were detected in both powders, with the value in the one freeze-dried with maltodextrin DE-15 being higher than in the one obtained by spray-drying, that is, 3.20 mg/g powder and 1.80 mg/g powder, respectively. Malic and citric acids, mild-tasting substances used as flavoring and preservative agents in foods, are antioxidant and show antimicrobial capacity [35].

The powdered beverages have high levels of total phenolic compounds, namely 3.20 ± 0.05 and 3.67 ± 0.07 mg GAE/g powder, similar in both drying methods. No significant differences were found in the content of flavonoids, i.e., 4.95 ± 0.02 and 5.13 ± 0.09 mg EQ/g powder, or in the content of condensed tannins, i.e., 1.13 ± 0.12 and 0.93 ± 0.09 mg EPB2/g powder. Anthocyanins, thermolabile pigments, which provide the red, blue, or purple color of fruits and the beverages obtained from them, were kept in a range of 0.141 ± 0.003 mg EC-3G/g in powders obtained by spray-drying and 0.381 ± 0.004 mg EC-3G/g in freeze-dried powders. The decrease in pigment content in the spray-dried beverages could be ascribed to the high temperature of the inlet gas to the equipment affecting the pigment stability, resulting in a 63% loss compared to the freeze-dried beverage [36,37]. Consequently, freeze-drying appears to be the most appropriate method for the drying process when microencapsulating the beverage in MD DE-15. In this way, the levels of anthocyanins and organic acids are better preserved.

After storing both powder beverages for 90 days at room temperature (25 °C) and protecting them from light, it was observed that the content of sugar, total phenolic compounds, flavonoids, anthocyanins, tannins, and organic acids remained constant.

### 2.3. Physicochemical and Flow Properties of the Powdered Beverage

The physicochemical properties of the powders (Table 3) are highly dependent on the drying method and operative conditions applied. The a_w_ values were higher than those of blackberry juice powders reported by Franceschinis et al. (2014) [38], and slightly higher than a powdered functional beverage formulated with *Solanum betaceum* fruits [39]. However, the values of both powders are low enough to ensure microbiological stability and to minimize deteriorating reactions (<0.3) [32]. The moisture content of both powders was lower than those reported by other berry-based powders [38].

The glass transition temperature permits predicting the behavior of the powders at different exposure temperatures. Low Tg values indicate that samples should be stored below that temperature to preserve physical properties. The Tg of the powder obtained by spray-drying is near room temperature, whereas freeze-dried powders showed much lower Tg. This behavior could be ascribed to the high concentration of sugars when adding fruit juice and honey to the beverages. Therefore, both powders should be stored at controlled refrigeration temperatures to ensure physical stability during storage, mainly in the freeze-dried products. Additionally, the hygroscopicity of both powders was high, around 26 g H_2_O/100 g DW, if the range reported for other spray-dried plant extracts or juices is considered [40]. Therefore, efficient packaging to remove ambient humidity will be essential to avoid stickiness and caking. However, the values of the Hausner ratio obtained and the Carr’s compressibility index values indicate very good flowability [30], which is acceptable for adequate powder handling.

There were no significant differences in the pH of both powders, and the total soluble solids (TSS) were similar (11 °Brix). As for solubility in water, both powders show quite a good one: 90%.

The color parameters indicated that the spray-dried powder is in the first quadrant of the CIELAB space, within the light-pink-color zone. The freeze-dried samples exhibit similar a* values but negative b* values, that is, within the region associated with dark purple hues in the fourth quadrant. This shift is mainly attributed to the greater retention of anthocyanin pigments after freeze-drying (Figure 3).

**Figure 4:**
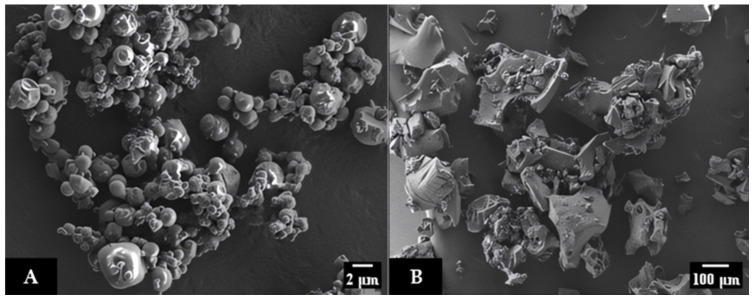
SEM micrographs of beverage spray-dried at 2500× (**A**) and freeze-dried at 800× (**B**).

Regarding particle size distribution, it was observed that spray-dried powders exhibited a median (D_50_) of 401 ± 27 μm, and a relatively broad size distribution (span values close to 2), which is consistent with the external morphology of the particles observed through electron microscopy (Figure 4A). A heterogeneous distribution of spherical particles was obtained after spray-drying, with a typical morphology for maltodextrin microcapsules. Small particles with a smooth surface, as well as larger ones with a certain degree of shrinkage, are formed because of the contraction of the liquid droplet; this is due to the rapid loss of moisture during the first stages of spray-drying; however, no cracks or pores were observed. Conversely, freeze-dried powders presented much bigger particles (D_50_ = 307.10 ± 12.62) of highly irregular shape and porous structure (Figure 4B). The operative variables used for the freezing step and further sublimation process determine the pore size distribution of the product. The porosity reached after the process, together with the conditions of the final pulverization necessary to obtain a particulate system, led to the morphology and particle size of freeze-dried powders observed in Figure 4B. Similar results were found by other authors for the freeze-drying of *Vaccinium myrtilus* juices encapsulated with MD [34]. Flow properties of a dry powder are related to interparticle adhesion, which is directly proportional to the contact area of particles, and inversely proportional to particle size. Additionally, particle morphology, i.e., porosity and roughness, and water content can also affect their flowability [41]. For instance, it should be expected that the roughness of particles hinders the interparticle approach, decreasing the adhesion forces and favoring fluidity, a result that seemed to occur in freeze-dried samples. The surface structure developed in these powders was found to have a major impact in comparison with particle shape and size distribution; in effect, better flowability was observed compared to spray-dried powders.

### 2.4. Functional Properties of Beverage Powders

#### Antioxidant Activity

In the present work, several methodologies [1,2,7,12] were used to determine the antioxidant capacity of the formulated beverages, as well as that of each ingredient. Both powdered beverages showed similar scavenging capacity of the ABTS^•+^ (Table 4). The antioxidant potency in both cases was similar to that of the liquid beverage (SC_50_ of 3.02 μg GAE/mL, Table 1). In all cases, the antioxidant potency of the beverage was higher than that of each ingredient alone, namely, blueberry juice (SC_50_ of 7.86 μg GAE/mL), honey 12 °Brix (SC_50_ of 8.40 μg GAE/mL), or *Z. punctata* extract (SC_50_ = 2.8 ± 0.02 µg GAE/mL).

After being stored for 90 days, both powdered beverages showed a similar antioxidant potency (SC_50_ µg GAE/mL 3.80 ± 0.33 spray-dried beverage and 3.98 ± 0.28 freeze-dried), shown in Table 4.

Both freeze-dried and spray-dried beverages showed scavenging capacity of hydrogen peroxide and hydroxyl radical with similar potency. The powders were also inhibitors of the XO enzyme, with IC_50_ values varying between 114.09 and 153.35 μg GAE/mL for spray-dried and freeze-dried powders, respectively. XO enzymes catalyze the oxidation of xanthine and hypoxanthine, derived from nucleic acid metabolism, into uric acid and superoxide anion. The overproduction of this acid leads to the appearance of hyperuricemia (gout disease). Some flavonoids, phenolic acids, and their derivatives have been reported to have inhibitory effects on the XO enzyme [42]. Most importantly, the formulated drink may inhibit xanthine oxidase, thereby blocking the generation of both uric acid and the superoxide anion, another free radical centered on the oxygen atom. Consequently, these non-alcoholic powdered beverages could be used as antioxidants to prevent oxidative stress produced by reactive oxygen species.

### 2.5. Inhibition of Enzymes Involved in Carbohydrate and Fat Metabolism

Obesity is considered a disorder of lipid metabolism, and the enzymes involved in this process could be selectively inhibited to develop drugs against this condition. Inhibition of the absorption of triglycerides and dietary carbohydrates through the inhibition of the enzymes pancreatic lipase, glucosidase, and amylase is considered a novel approach for the treatment of obesity [43]. Hence, it is important to consider the consumption of plant sources that provide specialized metabolites with this inhibitory activity. Table 5 shows that both the freeze- and spray-dried powders were inhibitors of the three enzymes assayed, being more active on the enzyme α-amylase than on lipase and α-glucosidase. The freeze-dried beverage was less active than the spray-dried beverage on α-glucosidase. The activity of both beverages on enzymes involved in lipid and sugar metabolism could be attributed to blueberry juice or *Z. punctata* extract; both have previously demonstrated the ability to reduce total cholesterol and the glycemic index in an in vivo obesity model [20,21,22,23,28]. The effect of *Z. punctata* extract on insulin resistance and blood pressure was also reported [20,21].

### 2.6. Sensorial Analysis

**Figure 5:**
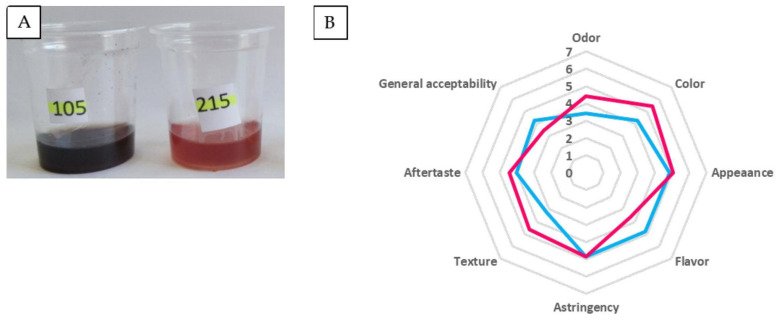
(**A**) Powdered beverages reconstituted in water presented to the panel of tasters for sensory evaluation. Sample 105: freeze-dried beverage; Sample 215: spray-dried beverage. (**B**)—Acceptance profile of beverage evaluated with the hedonic scale. Powdered beverage obtained by freeze-drying (blue line) and by spray-drying (pink line).

A sensorial analysis was performed to define the acceptance of the beverages with *Z. punctata* extract plus blueberry juice, and to state the difference in acceptability in relation to the drying method used. For this evaluation, a verbal hedonic scale was performed as sensory testing by using the hedonic or satisfaction scale. It is one of the most useful sensory methods to measure the degree of satisfaction that potential consumers have towards a product. It is easily understood by inexperienced consumers and entails minimal instruction. The beverage obtained by freeze-drying showed attributes with greater acceptance of flavor and general acceptability, with values of 4.8 and 4.2, respectively (Figure 5). Color appeared as the most accepted attribute in the spray-dried beverage (5.5). The sample that presented the greatest acceptance in terms of attributes, namely, color, odor, aftertaste, texture, and appearance, was the spray-dried sample, with an AF higher than 70%.

### 2.7. Toxicity

Mutagenicity was evaluated through the Ames test [44]. The mutagenicity ratio in all cases was less than 1.5 (Table 5 and Table 6); it would indicate the absence of mutagens that cause base substitution mutations (detected in TA100) and frameshift mutations (detected in TA98). Further, the acute toxicity of each of the ingredients and drinks was evaluated by using *A. salina* and *C. elegans* models. Three concentrations (250, 500, and 1000 µg GAE/mL) of each single ingredient (12 °Brix lemon honey, *Z. punctata* extract, and blueberry juice), as well as the powder formulations, were tested. At a concentration of up to 1000 µg GAE/mL, a 100% viability of *A. salina* nauplii was observed. So, none of the ingredients in the liquid beverage or the powders showed toxicity in the range of concentrations tested. In the in vivo test performed using *C. elegans* as a model organism, *Z. punctata* extract affected the viability of *C. elegans* larvae at concentrations higher than 500 µg GAE/mL. None of the other ingredients or the formulated beverages had any effect on the viability of *C. elegans* (Table 7). These results would indicate that powdered beverages would be safe for the consumer.

### 2.8. Microbiological Stability

The number of colony-forming units (CFU/g) of viable aerobic microorganisms, enterobacteria, or fungi and yeasts of the powdered beverages was null, and no significative change was observed during the 90 days of storage at 25 °C. The results reveal the microbiological stability of both beverages as expected, given the low water activity previously determined.

## 3. Materials and Methods

### 3.1. Reactives

Ethanol, phenol, NaCl, trichloroacetic acid, and H_2_O_2_ were purchased from Cicarelli (Santa Fe, Argentina). Maltodextrin DE 15 was purchased from Ingredion (Buenos Aires, Argentina). ABTS^•+^, 4-aminoantipyrine, horseradish peroxidase, Folin–Ciocalteu reagent, and quercetin were from Sigma-Aldrich, (St. Louis, MO, USA); 2-thiobarbituric acid, xanthine oxidase, 2-deoxy-D-ribose, α glucosidase, α amylase, lipase, xanthine oxidase, p-nitrophenyl-α-D-glucopyranoside, p-nitrophenyl palmitate, and p-nitrophenol were from Sigma-Aldrich (Darmstadt, Germany).

### 3.2. Juice Preparation

*Vaccinium corymbosum* fruits were purchased from a local market in Tucumán, Argentina, and the juice was made. Briefly, 1 kg of fully ripe fruits was processed in a blender coupled with a stainless-steel strainer (Philips Essence HR1357, Shanghai, China) to obtain 870 mL of juice. Then, it was used for the preparation of the beverages.

### 3.3. Zuccagnia punctata Extract

The aerial parts of *Z. punctata* Cav. were collected from northwest Argentina (26°35′33.3″ S 65°51′25.0″ W; 2329 m a.s.l.) and identified by botanist Dra. Soledad Cuello. The voucher specimen was deposited at the Herbarium of “Fundación Miguel Lillo”, Tucumán, Argentina (LIL 618078). The plant material (leaves and stems) was dried at 40 °C in a forced-air oven and then was ground in a Helix mill (Numak, F100 Power 1/2 HP-0.75 Kw, Brusque, Brazil) until constant weight. The powder (20 g) was macerated with 100 mL ethanol 5% for 30 min at 30 °C in an ultrasonic bath (Ultrasonic Washer “Arcano”). Then, the preparation was vacuum filtered to obtain *Z. punctata* extract 20% (*w*/*v*).

### 3.4. Honey 12 °Brix Preparation

Lemon honey was provided by beekeepers from Cooperativa Apícola Norte Grande, Tucumán, Argentina. Honey was mixed with water to obtain honey 12 °Brix according to the requirements of the Argentine food code for non-alcoholic beverages [29].

### 3.5. Beverage Formulation

*V. corymbosum* juice (27 mL), *Z. punctata* extract (9 mL), and honey 12 °Brix (sweetener) were used to prepare 100 mL of beverage. The percentage of blueberry juice and *Z. punctata* extract was selected according to the requirements of the Argentine Food Code for non-alcoholic beverages [29].

### 3.6. Microencapsulation of the Beverage

#### Spray-Drying and Freeze-Drying Processes

Maltodextrin DE 15 (15%) was added to the beverage, which was then dried by using a spray-dryer (Buchi Modelo B-290, Flawil, Switzerland) at 130 °C and 65 °C to let in and let out air temperatures, respectively. The liquid feed and drying air flow rate were 11 mL·min^−1^ and 600 L·h^−1^, respectively. The air flow in the drying chamber was 37 m^3^·h^−1^. The beverage was also flash frozen at −80 °C for 24 h. After that time, it was subjected to a freeze-drying process (freeze-dryer L-M10-A-E50-CRT, RIFICOR, Buenos Aires, Argentina) to eliminate the water content, thus obtaining a solid material, which was subjected to a grinding process in a propeller grinder (Numak F100, London, UK) to obtain the powdered beverage.

The dried beverages were vacuum sealed in tri-laminate bags, as follows: polyester outside, aluminum foil in the center, and food-grade polyethylene inside. They were then stored at room temperature.

### 3.7. Determination of Chemical Composition

#### 3.7.1. Total Polyphenol, Flavonoid, Hydrolyzed and Condensed Tannin, and Anthocyanin Quantification

The total phenolic compound contents of *Z. punctata* extract 20% (*w*/*v*), blueberry juice, and beverage were determined by using Folin–Ciocalteu reagent [45]. Total flavonoids were estimated by using the Woisky and Salatino method (1998) [46]. Results were expressed as μg of gallic acid equivalent per mL (μg GAE/mL) and quercetin equivalents per mL (μg QE/mL), respectively. Hydrolyzed and condensed tannins were quantified according to Carabajal et al. 2020 [5]. Data were expressed as mg procyanidin B_2_ per mL (mg PB_2_E/mL). Total anthocyanin content was analyzed by using the differential pH method according to Barros et al. 2010 [47]. Results were expressed as mg of cyanidin-3 glucoside equivalent per mL (mg C-3GE/mL).

#### 3.7.2. Reducing and Total Sugar Quantification

Reducing sugars and total sugar were determined by using the Somogyi–Nelson method [48,49] and phenol sulfuric method [50], respectively, in the *Z. punctata* extract 20% (*w*/*v*), the blueberry juice, the honey 12 °Brix, and the formulated beverage. The experiment was performed in triplicate and the results were expressed as glucose equivalent per mL (g GE/mL).

#### 3.7.3. HPLC-RID-DAD Analysis

Sucrose, glucose, and fructose contents, as well as malic and citric acids, were measured in the powders. The aqueous extracts were prepared by mixing 1 g of each powder with 12.5 mL distilled water, then constantly agitating it for 5 min. Then, each sample was filtered, and the remaining solid was again extracted by using the same procedure. Finally, more distilled water was added to reach a final volume of 25 mL. Soluble solids were determined, and samples were diluted to achieve 2 °Brix. Before HPLC analysis, the aqueous extracts were filtered with a 0.2 µm Nylon filter (Genbiotech SRL, Buenos Aires, Argentina) into a vial.

Glucose, fructose, and organic acids were separated by injecting 5 µL extract in an Agilent 1260 HPLC (Agilent Technologies, Santa Clara, CA, USA) equipped with an automatic injector (ALS) and two detectors: a diode array detector (DAD) for organic acid analysis and a refractive index detector (RID) for sugar analysis. Separation was performed by using a Hiplex H column (300 × 7.7 mm, 8 mm particle size, Agilent Technologies, Santa Clara, CA, USA) at 75 °C, and a mobile phase composed of 0.001 M H_2_SO_4_ with a flow rate of 0.4 mL/min (isocratic). For sucrose quantification, a ZORBAX carbohydrate column (150 × 4.66 mm, 5 µm particle size, Agilent Technologies, Santa Clara, CA, USA) and a mobile phase composed of 75% Acetonitrile and 25% Milli-Q water were used. The analysis was performed at 25 °C with a flow rate of 1 mL/min. Chromatograms were recorded at 214 nm and calibration curves were created by using the commercial standards with a high linearity (r^2^ > 0.999).

#### 3.7.4. Chalcones and Flavonoid Analysis by HPLC-DAD

The HLPC-DAD system used was Waters 1525 equipment with a Waters 1525 binary pump system, a manual injection valve (Rheodyne Inc., Cotati, CA, USA) with a 20 µL loop, a column thermostat compartment, and a Waters 2998 diode array detector. The analysis was performed at a temperature of 40 °C, using a 155 × 4.6 mm XBridge™ C18 (5 µm) column with a flow rate of 0.8 mL/min (Waters Corporation, Milford, MA, USA). The solvent system used for the separation of components from extracts was composed of solvent A (0.1% acetic acid in water) and solvent B (0.1% acetic acid in methanol) (conditions: 10–57% B from 0 to 45 min and kept at 100% B from 45 to 60 min). The flow rate was set at 0.5 mL/min. Data collection was carried out with Empower ^TM^ 2 software. The identification of phenolic compounds of *Z. punctata* extract and lemon honey was carried out by comparing the retention times and spectral data (220–600 nm) of each peak with those of standards, 2′,4′-dihydroxy-chalcone (DHC) and 2′,4′-dihydroxy-3-methoxychalcone (DHMC), from Indofine SRL, and hesperidin from Sigma Chemical Company. The quantification of different compounds was based on external calibration curves from available standards. Plots were built by comparison of area and concentration in the range of 1–500 ppm.

### 3.8. Biological Activities

The powdered beverages were reconstituted in water in a proportion of 10 g powder in 100 mL water and their functional properties were evaluated.

#### 3.8.1. Antioxidant Activity Assays

##### ABTS Radical Cation Decolorization

The antioxidant capacity assay of *Z. punctata* extract 20% (*w*/*v*), blueberry juice, honey 12 °Brix, and prepared beverages was performed by the improved ABTS^•+^ spectrophotometric method [51]. In this method, an ABTS^•+^ solution was mixed with different quantities of blueberry juice, *Z. punctata* extract, honey 12 °Brix, and beverage powder. Absorbance was recorded at 734 nm after 6 min. Results are expressed as SC_50_ values, with SC_50_ (μg GAE/mL) being defined as the concentration of phenolic compounds necessary to scavenge 50% ABTS free radicals.

##### Hydrogen Peroxide (H_2_O_2_) Scavenging

The H_2_O_2_ scavenging was assessed according to Fernando and Soysa (2015) [52]. The reaction mixtures contained phenol (12 mM), 4-aminoantipyrine 0.5 mM), H_2_O_2_ (0.7 mM), sodium phosphate buffer (84 mM) at pH 7, and different concentrations of the blueberry juice, *Z. punctata* extract, and powdered beverage. They were kept at 35 °C for 20 min. Then, horseradish peroxidase (0.1 U/mL) was added, and they were incubated at 37 °C for 30 min. The absorbance was measured at 504 nm. Results are expressed as SC_50_ values in μg GAE/mL.

##### 2-deoxy-D-ribose Degradation

To evaluate the hydroxyl radical scavenging capacity of the beverage and of each ingredient in particular, the 2-deoxy-D-ribose degradation assay was applied as described by Chobot [53]. After the Fenton reaction, 250 μL of 2-thiobarbituric acid (1% *w*/*v*) dissolved in trichloroacetic acid (3% *w*/*v*) was added to each vial to detect malondialdehyde (MDA). The tubes were vortexed and heated at 100 °C for 20 min. The reaction was halted by transferring the tubes into an ice-water bath. Absorbance was determined at 532 nm. Results are expressed as SC_50_ values in μg GAE/mL.

##### Xanthine Oxidase 

The effects of different aliquots of extracts, juice, and beverage on the activity of xanthine oxidase (0.003 U) were determined spectrophotometrically at 290 nm (Microplate Reader Thermo Scientific Multiskan GO, Vantaa, Finland) by measuring the synthesis of uric acid from xanthine as substrate (60 μL, 1 mM) [53]. The reaction mixture (120 μL) was incubated at 25 °C for 30 min [54]. Results are expressed as IC_50_ values in μg gallic acid per mL (μg GAE/mL). IC_50_ (μg GAE/mL) was defined as the concentration of phenolic compounds necessary to inhibit 50% enzyme activity.

#### 3.8.2. Inhibitory Activity of Enzymes Involved in Carbohydrate and Fat Metabolism

##### α-Amylase Inhibition

The effect of the extract, juice, or drink on the activity of the α-amylase enzyme was evaluated by using the Amilokit test (Wiener Lab Group, Rosario, Argentina, Cat No. 1021001) as reported by Costamagna et al. [54].

##### α-Glucosidase Inhibition

The assay was performed according to Costamagna et al. [54] with some modifications, by using p-nitrophenyl-α-D-glucopyranoside as a substrate. In the reaction, 160 µL of 0.1 M sodium phosphate buffer (pH 6.9) was contacted with 5 µL of 5.46 U/mL enzyme and the extract, juice, or beverage at 4 °C for 10 min. Subsequently, 5 mM p-nitrophenyl-α-D-glucopyranoside was added and incubated for 15 min at 37 °C. Absorbance was measured at 420 nm. IC_50_ values were calculated by interpolating dose–response curves.

##### Lipase Inhibition

The lipase enzyme activity was measured according to Costamagna et al. [54] by means of the enzymatic hydrolysis of p-nitrophenyl palmitate to p-nitrophenol.

### 3.9. Physicochemical Properties

#### 3.9.1. Moisture, Soluble Solid Content, and Water Activity

The powdered beverage was characterized according to AOAC methods [55]: moisture, soluble solids, and pH. Water activity (a_w_) was determined at 25 ± 1 °C by using an electronic dew-point water activity meter, Aqualab Series 3 TE (Decagon Devices, Pullman, WA, USA).

#### 3.9.2. Glass Transition Temperature (Tg)

Glass transition temperature was determined by differential scanning calorimetry (DSC; onset values) by using a DSC 822e Mettler Toledo calorimeter (Schwerzenbach, Switzerland) [56].

#### 3.9.3. Superficial Color

Superficial color was determined (CIELAB color space) with a photo colorimeter model CR 400 (Minolta, Tokyo, Japan) by using illuminant C and a 2° observer angle. L* (brightness/darkness), a* (redness/greenness), and b* (yellowness/blueness) values were recorded. The numerical values were converted into “chroma” (C*_ab_) and “hue angle” (h_ab_) parameters.

#### 3.9.4. Solubility

Powder samples (0.5 g) were dissolved in 50 mL distilled water and then centrifuged (BioAmerican Science, Buenos Aires, Argentina) at 3000× *g* for 5 min. Ten mL of supernatant was transferred to a glass capsule and dried in an air oven at 105 °C until constant weight. Solubility (%) was calculated by weight difference [40].

#### 3.9.5. Bulk and Compacted Density, Flowability (Carr Index), and Cohesiveness (Hausner Ratio)

Bulk and compacted density (g/mL) was determined according to Gallo et al. [57] with some modifications. The Hausner ratio (H) was related to the powder cohesiveness, where levels below 1.2 are considered low, between 1.2–1.4, intermediate, and above 1.5, high. Carr’s compressibility index (CI) was determined. The following scale was used: (a) CI = 10 excellent; (b) 11 < CI < 15 good; (c) 16 < CI < 20 fair; (d) 21 < CI < 25 acceptable; and (e) 26 < CI < 31 poor.

#### 3.9.6. Hygroscopicity

Hygroscopicity was determined in a closed desiccator at 20 °C, containing a saturated solution of NaCl (75% RH). The samples were weighed for twenty days, and hygroscopicity was expressed as the average of grams of adsorbed water per 100 g of dry matter (g aw/100 g dm).

### 3.10. Particle Morphology

Scanning electron microscopy (SEM) was applied to study the microstructural characteristics of spray-dried particles by using a Zeiss microscope Supra 40 (Carl Zeiss, Oberkochen, Germany). Samples were placed on an aluminum support using conductive carbon doubled-sided adhesive tape and coated by using a sputter coating machine model 108 (Cressington Scientific Instruments, Watford, UK) with gold nanoparticles. Micrographs were taken by using an acceleration voltage of 10.00 kV at a magnification range between 800 and 2500×.

Particle size distribution was measured according to Sette et al. 2022 [41], with a laser light diffraction instrument LA-950 V2 (Horiba, Kyoto, Japan) and using the dry powder method. The distribution width was characterized in terms of span index: span = (D_90_ − D_10_)/D_50_ where D_90_, D_50_, and D_10_ are the diameters for which the mass of the particle population is below 90%, 50%, and 10%, respectively. Values of the span close to 1 indicate a narrower particle distribution.

### 3.11. Toxicity

#### 3.11.1. Acute Toxicity

Different concentrations of beverage (250–1000 µg GAE/mL) were used to determine its acute toxic effect by using the *Artemia salina* microplate assay [58]. Positive controls with potassium dichromate (Sigma-Aldrich, Darmstadt, Germany) (250–1000 µg GAE/mL) were assayed. After 24 h exposition, the number of dead shrimps in each well was recorded.

#### 3.11.2. *Salmonella* Typhimurium Assay

Toxicity Assay. To examine the effects on the viability of *Salmonella* Typhimurium strains TA98 and TA100, a concentration range of extract, i.e., 125–500 µg GAE/plate, was added to overnight-cultured *Salmonella* Typhimurium strains TA98 or TA100 (0.1 mL). The mixture was poured onto nutrient agar (Britania, Ciudad Autónoma de Buenos Aires, Argentina) plates, which were incubated at 37 °C for 2 days, and the number of colonies was counted. The beverages were then tested for their mutagenic potency exclusively in the nontoxic concentration range [59].

Mutagenicity Assay. *Salmonella* Typhimurium strains TA98 and TA100 were used to evaluate the possible mutagenic effect of the beverage (500 µGAE/plate) [59]. The 4-nitro-o-phenylenediamine (Sigma Aldrich, St. Louis, MO, USA) reagent (4-NPD, 5 µg/plate) was used as a positive control. All experiments were analyzed in triplicate with at least two replicates. The results were expressed as the number of revertants/plate, and the mutagenicity ratio (MR), that is, the ratio between the number of test plate revertants (induced revertant, IR) and the number of revertants on the control plate (spontaneous revertant, ER), MR = IR/ER, was also calculated. The samples were considered mutagenic when the revertant average number was at least twice as much or higher than the spontaneous revertants, or if the MR was above two [59].

#### 3.11.3. *Caenorhabditis elegans*

Toxicity assays were performed in sterile 24-well polycuvettes according to Solis and Petrascheck [60], with some modifications. Ten µL of a nematode solution containing 50 individuals was transferred to each well previously seeded with the different concentrations of the samples to be tested (250, 500, and 1000 µg/mL) and incubated at 25 °C. Controls were performed without the samples and with DMSO. The count of live and dead individuals was performed after 24 h under a magnifying glass [61].

### 3.12. Sensory Analysis

The sensory evaluation of the powdered beverage was performed by using the verbal hedonic scale. The beverages were reconstituted (1 g/10 mL, *w*/*v*) in drinking water, served in transparent glasses of 5 cm diameter, and kept at 10 °C. All samples were evaluated under white light illumination. Water and unsalted crackers were provided to clear the taste between samples. Hedonic sensory testing was conducted by an untrained panel (*n* = 52) with an age range between 25 and 65, most of them female, to rate acceptability using a 7-point verbal hedonic scale, which ranged from “I dislike it very much” to “I like it very much”, with an intermediate point of neither “I like it” nor “I dislike it”, to evaluate the drinks separately in 8 attributes: general pleasantness, color, appearance, aroma, flavor, texture, astringency, and aftertaste. For evaluation, a minimum of 15 mL of each sample per evaluator was poured into identical containers labeled with 3-digit numerical codes. To verify the acceptability of beverages, an acceptability factor (AF) was calculated [62] with respect to each attribute analyzed:

AF = A × 100 × B^−1^

where A is the average value obtained for each attribute and B is the maximum value for each attribute.

### 3.13. Stability Assays over Time

Microbiological, phytochemical, and functional stability tests were performed at zero time and at three months of storage, at 25 °C.

In the microbiological stability test, the number (CFU/g) of viable aerobic microorganisms, enterobacteria, and fungi and yeasts of the powdered beverage preserved at 25 °C was determined by the serial dilution method. Each sample was homogenized with 9 mL of sterile physiological solution. From this suspension, serial dilutions were made and 100 μL of each dilution was plated on different culture media (Plate Count Agar, Mac Conkey Agar, and Molds & Yeasts Agar). Suitable aliquots obtained were taken at different times and inoculated in the culture media. The number of CFU/g of beverage was determined at 0 (initial time) and 1, 2, and 3 months [63].

For the phytochemical stability test, the content of total phenolic compounds, flavonoids, and anthocyanins was determined according to the methodology described in Section 3.7.1. The functional stability of both powdered drinks was evaluated through the antioxidant activity of the ABTS radical cation, described in Section ABTS Radical Cation Decolorization.

### 3.14. Statistical Analysis

For the statistical analysis of the data, the Tukey test was applied, with a level of significance *p* > 0.05, by using the statistical package InfoStat V1.1 [64].

## 4. Conclusions

Functional beverages that incorporate native plant extracts as ingredients from different regions, e.g., *Z. punctata* from Argentina, are excellent options to promote the human welfare of entire populations and, at the same time, encourage the development of regional economies. The formulations developed with *Z. punctata* and *V. corymbosum* contain microparticles that have high water solubility, and display traits that make them suitable for handling, transport, and storage. Therefore, the present study highlights the promising potential of botanicals as an additional source of bioactive compounds. Further studies are necessary to confirm the functionality of these beverages as antioxidants and hypoglycemic and lipid-lowering agents by in vivo models.

## Figures and Tables

**Table 1:** Chemical composition of beverage formulated with *Z. punctata* extract and blueberry juice.

	Beverage
	Phytochemicals
Total phenolic compounds (mg GAE/mL)	0.92 ± 0.01
Total flavonoids (mg QE/mL)	1.34 ± 0.02
Anthocyanins (mg C-3GE/mL)	0.92 ± 0.09
	Nutritional components
Total sugars (mg GE/mL)	125.62 ± 4.02
Reducing sugars (mg GE/mL)	115.32 ± 4.57
	Antioxidant activity
ABTS^•+^SC_50_ (µg GAE/mL)	3.02 ± 0.01

GAE: gallic acid equivalent; QE: quercetin equivalent; C-3GE: cyanidin 3-glucoside equivalent; GE: glucose equivalent. ABTS^•+^: ABTS radical cation. Values are reported as mean ± standard deviation of triplicates.

**Table 2:** Chemical characterization of spray-dried beverage and freeze-dried beverage with maltodextrin DE-15.

	Spray-Dried Beverage	Freeze-Dried Beverage
Glucose mg/g powder	50.00 ± 0.10 ^a^	51.90 ± 0.20 ^a^
Fructose mg/g powder	63.00 ± 0.50 ^a^	65.80 ± 0.10 ^a^
Sucrose mg/g powder	ND	ND
Total proteins (mg BSA/g powder)	1.82 ± 0.11 ^a^	1.70 ± 0.11 ^a^
Citric acid (mg/g powder	2.30 ± 0.01 ^a^	3.20 ± 0.02 ^b^
Malic acid (mg/g powder)	1.10 ± 0.08 ^a^	1.80 ± 0.08 ^b^
Total phenolic (mg GAE/g powder)	3.20 ± 0.05 ^a^	3.67 ± 0.07 ^a^
Flavonoids (mg QE/g powder)	4.95 ± 0.02 ^a^	5.13 ± 0.09 ^a^
Condensed tannins (mg PB_2_E/g powder)	1.13 ± 0.12 ^a^	0.93 ± 0.09 ^a^
Anthocyanins (mg C-3GE/g powder)	0.141 ± 0.003 ^a^	0.381 ± 0.004 ^b^

BSA: bovine serum albumin equivalent; GAE: gallic acid equivalent; QE: quercetin equivalent; PB_2_E: procyanidin B2 equivalent; C-3GE: cyanidin 3-glucoside equivalent. ND: non detected. Values are reported as mean ± standard deviation of triplicates. Different letters in the same row indicate significant differences between formulations according to Tukey’s test (*p* ≤ 0.05).

**Table 3:** Physicochemical characterization of spray-dried and freeze-dried beverages of *Z. punctata* extract and blueberry juice.

Properties	Spray-Dried Beverage	Freeze-Dried Beverage
a_w_	0.309 ± 0.001 ^a^	0.289 ± 0.007 ^a^
Moisture (%)	4.53 ± 0.05 ^a^	4.84 ± 0.02 ^a^
pH	5.32 ^a^	5.22 ^a^
Total soluble solid (°Brix at 25 °C)	11 ± 0.1 ^a^	11 ± 0.1 ^a^
L*	63.5 ± 0.4 ^a^	40.4 ± 0.6 ^b^
a*	10.53 ± 0.11 ^b^	12.1 ± 0.5 ^a^
b*	1.66 ± 0.16 ^a^	−1.22 ± 0.10 ^b^
Chroma	10.66 ± 0.13 ^a^	12.2 ± 0.5 ^a^
Hue angle	8.9 ± 0.8 ^a^	354.0 ± 0.4 ^b^
Bulk density (g/mL)	0. 502 ± 0.003 ^b^	0.713 ± 0.008 ^a^
Compacted density (g/mL)	0.557 ± 0.003 ^b^	0.77 ± 0.02 ^a^
Hausner ratio	1.111 ± 0.001 ^a^	1.07 ± 0.03 ^a^
Carr index	10.000 ± 0.001 ^a^	6.7 ± 0.3 ^b^
T_g_ (°C)	23.79 ± 1 ^a^	13.76 ± 1 ^b^
Hygroscopicity (g H_2_O/100 g DW)	26.4 ± 0.5 ^a^	26.3 ± 0.2 ^a^
Solubility	90.60 ± 0.14 ^b^	90.8 ± 0.4 ^a^
D_10_ (µm)	84.8 ± 0.3 ^b^	102.8 ± 1.8 ^a^
D_50_ (µm)	401 ± 27 ^a^	307 ± 13 ^b^
D_90_ (µm)	732 ± 95 ^a^	704 ± 102 ^a^
Span	1.62	1.96

a_w_: water activity. L*: lightness, a* and b*: chromaticity. T_g_: transition temperature. D: particle size. Values are reported as mean ± standard deviation of triplicates. Different letters in the same line indicate significant differences between formulations according to Tukey’s test (*p* ≤ 0.05).

**Table 4:** Antioxidant capacity of the powdered beverage.

		Antioxidant Activity			
	ABTS^•+^ T_0_	ABTS^•+^ T_90_	H_2_O_2_	HO•	XO
	SC_50_ (µg GAE/mL)		SC_50_ (µg GAE/mL)	SC_50_ (µg GAE/mL)	IC_50_ (µg GAE/mL)
Spray-dried beverage	3.56 ± 0.32 ^a^	3.80 ± 0.33 ^a^	33.60 ± 0.28 ^a^	35.88 ± 0.28 ^a^	114.09 ± 0.49 ^b^
Freeze-dried beverage	3.76 ± 0.04 ^a^	3.98 ± 0.28 ^a^	29.16 ± 0.2 ^a^	36.90 ± 0.42 ^a^	153.35 ± 2.47 ^a^

XO: xanthine oxidase; GAE: gallic acid equivalent. SC_50_: scavenging concentration 50% of free radicals; IC_50_: inhibitory concentration 50% of XO activity. ABTS^•+^: ABTS cation radical; H_2_O_2_: hydrogen peroxide; HO•: hydroxyl radical. Values are reported as mean ± standard deviation of triplicates. Different letters in the same column indicate significant differences between formulations according to Tukey’s test (*p* ≤ 0.05). T_0_: spray-dried and freeze-dried beverages recently prepared. T_90_: spray-dried and freeze-dried beverages maintained for 90 days.

**Table 5:** Effect of beverage powders on enzymes involved in carbohydrate and fat metabolism.

	α-Glucosidase	α-Amylase	Lipase
	IC_50_ (µg GAE/mL)	IC_50_ (µg GAE/mL)	IC_50_ (µg GAE/mL)
Spray-dried beverage	26.03 ± 2.39 ^b^	3.82 ± 0.26 ^a^	23.89 ± 0.09 ^a^
Freeze-dried beverage	42.16 ± 1.68 ^a^	2.97 ± 0.06 ^a^	27.19 ± 0.05 ^a^

IC_50_: inhibitory concentration of 50% enzymatic activity. GAE: gallic acid equivalent. Values are reported as mean ± standard deviation of triplicates. Different letters in the same column indicate significant differences between formulations according to Tukey’s test (*p* ≤ 0.05).

**Table 6:** Mutagenicity assessment. Number of revertants/plate and mutagenicity ratio (MR) in *S*. typhimurium TA98 and TA100 with different doses of each sample.

Beverage/Ingredients	µg GAE/Plate	TA 98	TA 100	MR TA 98	MR TA 100
*Z. punctata* extract	125	30 ± 4	97 ± 3	0.90	0.91
	250	33 ± 3	99 ± 4	0.97	0.93
	500	35 ± 4	99 ± 3	1.04	0.92
Blueberry juice	125	37 ± 2	97 ± 1	1.10	0.91
	250	32 ± 3	109 ± 3	0.94	1.02
	500	31 ± 6	91 ± 4	0.93	0.85
Lemon honey	125	32 ± 1	102 ± 3	0.94	0.95
	250	33 ± 5	108 ± 2	0.97	1.01
	500	31 ± 4	107 ± 4	0.93	1.00
Freeze-dried beverage	125	36 ± 3	203 ± 9	0.96	0.95
	250	35 ± 5	205 ± 9	0.93	0.96
	500	36 ± 2	194 ± 2	0.97	0.91
Spray-dried beverage	125	33 ± 5	202 ± 6	0.88	0.95
	250	35 ± 2	184 ± 8	0.95	0.87
	500	38 ± 4	197 ± 3	1.03	0.92
Positive control		1494 ± 109	989 ± 171	40.37	4.64
Negative control		37 ± 2	213 ± 16		

µg GAE/plate: µg of gallic acid equivalents/plate. MR: mutagenicity ratio. Values are presented as mean ± standard deviation of triplicates. Positive control: 4-nitro-phenylenediamine. Negative control: DMSO.

**Table 7:** Toxicity test on *C. elegans* nematodes.

	µg GAE/mL	% Viability
*Z. punctata* extract	250	90.1 ± 2.1
	500	72.5 ± 1.2
	1000	45.4 ± 2.2
Blueberry juice	250	96.6 ± 0.7
	500	94.2 ± 0.8
	1000	94.9 ± 2.3
Lemon honey	250	97.1 ± 1.3
	500	99.0 ± 0.9
	1000	96.4 ± 3.2
Freeze-dried beverage	250	93.3 ± 1.7
	500	92.1 ± 2.1
	1000	93.0 ± 1.5
Spray-dried beverage	250	95.0 ± 1.6
	500	94.8 ± 1.9
	1000	95.3 ± 2.0
Negative control		90.0 ± 2.9

µg GAE: µg of gallic acid equivalents. Values are presented as mean ± standard deviation of triplicates. Negative control: Buffer M9.

## Data Availability

Data are contained within the article.
